# The symptoms evolution of long COVID‑19 (SE-LC19): a new patient-reported content valid instrument

**DOI:** 10.1186/s41687-024-00737-5

**Published:** 2024-08-09

**Authors:** Diana Rofail, Selin Somersan-Karakaya, Eleftherios Mylonakis, Julia Y. Choi, Krystian Przydzial, Sarah Marquis, Yuming Zhao, Mohamed Hussein, Thomas D. Norton, Anna J. Podolanczuk, Gregory P. Geba

**Affiliations:** 1 Regeneron Pharmaceuticals, Inc., 1 Rockwood Road, Sleepy Hollow, NY 10591 USA; 2 Infectious Diseases Division, Warren Alpert Medical School of Brown University, Providence, RI USA; 3 Department of Medicine, Houston Methodist Hospital, Houston, TX USA; 4 Modus Outcomes, Cambridge, MA USA; 5 Weill Cornell Medical College, New York, NY USA

**Keywords:** Long COVID, Long-haul COVID, Long haulers, Patient-reported outcomes, Patient-reported outcome measures, Post-COVID conditions, Qualitative, Questionnaire, Symptoms

## Abstract

**Background:**

The field of long COVID research is rapidly evolving, however, tools to assess and monitor symptoms and recovery of the disease are limited. The objective of the present study was to develop a new patient-reported outcomes instrument, the Symptoms Evolution of Long COVID‑19 (SE-LC19), and establish its content validity.

**Methods:**

The 40-item SE-LC19 instrument was developed based on patient-relevant empirical evidence from scientific literature and clinical guidelines that reported symptoms specific to long COVID. A 2-part mixed-method approach was employed. Part 1: Qualitative interviews with a purposive sample of 41 patients with confirmed long COVID were conducted for the content validation of SE-LC19. During cognitive debriefing interviews, patients were asked to describe their understanding of the instrument’s instructions, specific symptoms, response options, and recall period to ensure its relevance and comprehensiveness. Five clinicians of different medical specialties who regularly treated patients with long COVID were also interviewed to obtain their clinical expert opinions on SE-LC19. Part 2: Exploratory Rasch Measurement Theory (RMT) analysis was conducted to evaluate the psychometric properties of the SE-LC19 data collected during the interviews.

**Results:**

Overall, patients reported that the instructions, questions, recall period, and response options for SE-LC19 were comprehensive and relevant. Minor conceptual gaps reported by patients captured nuances in the experience of some symptoms that could be considered in future studies. Some patients suggested a revision of the recall period from 24 h to 7 days to be able to capture more symptoms given the waxing and waning nature of some symptoms. Clinicians found the instrument comprehensive with minimal suggestions regarding its content. Exploratory RMT analyses provided evidence that the SE-LC19 questionnaire performed as intended.

**Conclusion:**

The present mixed-methods study in patients with confirmed long COVID supports the content validity and applicability of the SE-LC19 instrument to evaluate the symptoms of patients with long COVID. Further research is warranted to explore the psychometric properties of the instrument and refine a meaningful and robust patient-relevant endpoint for use in different settings such as clinical trials and clinical practice to track the onset, severity, and recovery of long COVID.

**Supplementary Information:**

The online version contains supplementary material available at 10.1186/s41687-024-00737-5.

## Introduction

As patients recovered from the acute phase of coronavirus disease 2019 (COVID-19) infection, many reported difficulties in returning to their previous baseline function, despite negative respiratory viral test results [1]. Furthermore, it became clear that some patients were experiencing a broad spectrum of additional symptoms, affecting one or several organ systems, that they had not experienced previously [1–4]. Long COVID has become a commonplace term used to describe a collection of symptoms that persist for at least 4 weeks after contracting COVID-19; however, our knowledge of the disease is still expanding and the true impact that long COVID has on patients and the healthcare system is only starting to emerge [5]. Health bodies such as the Centers for Disease Control and Prevention (CDC) and the National Institute for Health and Care Excellence (NICE), refer to symptoms that cannot be explained by an alternative diagnosis and which persist for longer than 4 weeks after acute infection with SARS CoV-2, as long COVID [3, 4]. Terms such as post-COVID-19 syndrome (PCS) or post-COVID conditions (PCC) may also be used to refer to long COVID, but usually when symptoms develop or persist for 12 weeks after the initial infection. Long COVID encompasses a wide range of seemingly unrelated symptoms. To date over 200 symptoms have been reported [6], and the condition itself can last for months or years [4], with symptoms that may fluctuate over time [7, 8]. The CDC estimate that 40% of adults in the United States reported having COVID-19 and 19% of these are currently still having symptoms, approximately 7.5% of the population [9]. Data from the CDC also found that younger adults and females are more likely to develop long COVID than older adults and males, respectively [9].

Previously, we reported the development of a conceptual model in long COVID that provides a visual representation of information that spontaneously emerges when speaking to people with long COVID, which is organized in a meaningful way by proximal and distal concepts of interest [10]. Results from the concept elicitation interviews indicated that the patient experience of long COVID negatively impacts patients’ daily lives. In addition to experiencing upper and lower respiratory tract symptoms, altered or loss of taste and/or smell, and systemic and gastrointestinal symptoms, patients also experience profound neuro-cognitive symptoms not typically reported by those in the acute stages of COVID-19. These symptoms included numbness, ringing in ears, haziness, confusion, forgetfulness, memory problems, brain fog, concentration, difficulties finding the right word, and challenges with fine motor skills that can be pertinent for several months. These symptoms in turn negatively impacted patients’ broader health-related quality of life, including being able to go about their activities of daily living, physical and emotional health, social/leisure activities, work, and productivity.

With the growing body of evidence for long COVID, there was an acknowledgement of the importance of generating reliable and valid patient experience data using a patient-reported outcome measure that captures patients’ self-reported symptoms, as well as a need to ensure interpretable outcomes that can support evaluation of benefits (efficacy) and risks/harms (safety) of an intervention in future clinical studies. Given that at the time of this study, there were no specific instruments/tools available to assess patient-reported outcomes for long COVID, the Symptoms Evolution of COVID-19 (SE-C19) instrument, originally developed in acute COVID-19, was used to form the foundations of the Symptoms Evolution of Long COVID-19 (SE-LC19) [11, 12]. Closer examination of long COVID symptoms reported in scientific literature and those reported by relevant clinical guidelines (i.e., the CDC [4], World Health Organization [WHO] [13], and NICE) indicated that the symptoms of the original SE-C19 appeared to be relevant to people with long COVID [3]. The SE-LC19 followed good measurement principles as outlined in the US Food and Drug Administration (FDA) Patient-Reported Outcome guidance [14] and the Patient-Focused Drug Development guidance documents [15–19]. It included 40 items (Table S1), where patients are required to rate their symptoms at their worst moment during the last 24 h on a 4-point severity scale (no symptom, mild, moderate, or severe).

During the initial phase of development of the SE-LC19 instrument (reported previously) we sought to gain an in-depth understanding of patients’ experience of long COVID through a series of literature searches and the development of a preliminary concept model [10]. In this paper, we summarize our findings from the second stage development of SE-LC19, evaluation of the instrument’s content validity, and preliminary modern psychometrics of the instrument in patients with long COVID.

## Methods

There were two parts to this mixed-methods study. The first was a qualitative aspect that involved cognitive debriefing of the SE-LC19 (part 1) in a “think aloud” process to explore the content validity of the instrument and the second was performing preliminary modern psychometrics of the SE-LC19 (part 2). All study documents were submitted to WCG (WIRB [Western Institutional Review Board] and Copernicus Group) Institutional Review Board (WCG-IRB) and approved [IRB tracking #20214272]. The present study was conducted in accordance with the “Consolidated Criteria for Reporting Qualitative Research” guidelines [20].

### Participants inclusion and exclusion criteria

Adults (≥18 years) with a clinically confirmed diagnosis of long COVID, at least one risk factor for severe COVID-19, a positive polymerase chain reaction test for SARS-CoV2 at least 180 days prior to the enrolment and experiencing long COVID symptom(s) that could not be explained by an alternative diagnosis were invited to participate. Eligible patients also had to be willing and able to participate in a 60-min audio-recorded telephone or online interview in English. Full details of the participant eligibility criteria can be found in the Supplementary Methods. All patients provided electronic informed consent for their participation in this study. Demographic and self-reported health information was collected from all patients.

In addition to patients with long COVID, five clinicians active in the field of post-COVID-19 care were invited to partake in in-depth interviews including providing their thoughts on the SE-LC19. Clinicians were eligible to participate in an interview if they were regularly seeing or treating patients with long COVID in the US (more than five patients a week); and were willing to participate in a 60-min audio-recorded telephone interview in English. Clinicians who only treated patients residing in an institutional setting, such as a nursing home, were not included.

### Sampling

Due to the heterogeneity of symptoms experienced in long COVID, sampling was defined to target a wide range of symptoms and patients. A purposive sample of patients was originally identified through Regeneron Pharmaceutical Inc.’s clinical trial sites and subsequently extended to the real-world through two independent, global, healthcare research firms: Rare Patient Voice and PRC Corporation Market Research. Sampling was defined to target a population experiencing long COVID symptoms and to recruit a sample of up to 50 patients. The final sample size was determined by saturation based on good research practice [21, 22] and requirements by the FDA for establishing content validity [23].

Although, there were no pre-defined sample quotas set for the five clinicians, the recruitment process was based on a diverse representation of professions (such as general practitioner, nurse), specialties, and treatment settings where patients with long COVID received treatment (Table S2).

### Concept to item mapping

The SE-LC19 questionnaire contains 40 items (individual symptoms; Table S1) and these items were mapped to the subdomains of the previously developed conceptual model [10].

### Part 1: cognitive debriefing interviews procedure

Detailed patient interviews (~60 min each) were conducted via Microsoft Teams by four experienced qualitative researchers who received specific training for this study. All interviews were audio recorded. The cognitive debriefing interview consisted of a “think aloud” debriefing of the SE-LC19, which allowed the participants to verbalize their thoughts and provide a response to each of the SE-LC19 items, response options, and recall period to determine their relevance, comprehensiveness, and comprehensibility.

More specifically, patients were asked to describe their understanding of the SE-LC19 instructions and specific symptoms listed to ensure that the intended connotation of each item was consistent with the patient’s interpretation or assigned meaning. Patients were also asked for their understanding of the response options for symptoms that they had reported experiencing in the past 24 h. To ensure the relevance and comprehensiveness of the content, patients were also asked if other symptoms should be added or if any present symptoms should be removed.

### Qualitative data analysis

Cognitive debriefing analysis is designed to identify and categorize the issues spontaneously reported by patients and those specifically probed by the interviewer. Cognitive debriefing interviews were coded using multiple levels of codes containing information on the clarity, comprehension, and interpretation of the items, response options, and instructions. The first transcript was independently coded by three researchers. Coding inconsistencies were discussed and reconciled. The three researchers continued to meet regularly to adjust coding guidelines where necessary. Patient feedback collected during cognitive debriefing interviews was compiled into cognitive debriefing summary tables, which included patients’ comments for each item. Any issues related to understanding the item content or providing a response were recorded. Feedback on the instructions, format, and layout of the questionnaire was also recorded. Additional sub-domains relevant to the variables under measurement, but not directly measured by the existing item list, were also identified.

### Part 2: modern preliminary psychometrics

Rasch Measurement Theory (RMT) is a family of statistical models and techniques conducted to evaluate the psychometric properties of instrumental data. In this study, exploratory RMT analyses were performed to provide a preliminary understanding of the following psychometric properties of the SE-LC19: targeting, the measurement continuum, and sample measurement. Exploratory RMT analysis was performed using RUMM2030 software. Details of the exploratory RMT analyses are provided in the Supplementary Methods.

## Results

### Patient characteristics

Patient interviews were conducted between September 30, 2021 and May 12, 2022. The present analysis included 41 patients, of which 18 (44%) were recruited through Regeneron Pharmaceuticals Inc.’s clinical trials and 23 (56%) were recruited through recruitment agencies (Table 1). The mean (standard deviation [SD]) age of the patients was 53.6 (10.2) years and the majority were female (85%) and White (73%). Self-reported patient health information was collected at the time of the interview and is summarized in Table 1. Most patients reported good (34%) or fair (37%) general health at the time of the interview. The mean (SD) time between the start of COVID-19 symptoms and the interview was 12.1 (5.9) months.

**Table 1:** Overview of patient sample characteristics

Variable^a^	All (N = 41)	Clinical trials (n = 18)	Recruited (n = 23)
Age, years, mean (SD)	53.56 (10.24)	56.44 (11.01)	51.30 (9.20)
Sex, n (%)			
Female	35 (85.4)	14 (77.8)	21 (91.3)
Male	6 (14.6)	4 (22.2)	2 (8.7)
Race, n (%)^b^			
White/Caucasian	30 (73.2)	12 (66.7)	18 (78.3)
Black/African American	9 (21.9)	4 (22.2)	5 (21.7)
American Indian/Alaskan	1 (2.4)	0 (0.0)	1 (4.3)
Asian	1 (2.4)	0 (0.0)	1 (4.3)
Other	2 (4.9)	1 (5.5)	1 (4.4)
Prefer not to answer	1 (2.4)	1 (5.5)	0 (0.0)
Self-reported health information at the time of the interview			
General health ratings, n (%)			
Excellent	3 (7.3)	2 (11.1)	1 (4.3)
Very good	3 (7.3)	3 (16.7)	0 (0.0)
Good	14 (34.1)	7 (38.9)	7 (30.4)
Fair	15 (36.6)	5 (27.8)	10 (43.5)
Poor	6 (14.6)	1 (5.5)	5 (21.7)
**Number and duration of symptoms**			
Number of months since symptoms began, mean (SD)	12.15 (5.87)	7.72 (2.11)	15.61 (5.53)
Number of symptoms reported per patient, mean (SD)	7 (4.73)	6 (4.48)	8 (4.87)
**Time between hospitalization due to COVID-19 and interview (months)**			
Not hospitalized, n (%)	21 (51.2)	8 (44.4)	13 (56.5)
Mean (SD)	10.4 (4.95)	7.00 (6.67)	13.8 (5.05)
Self-re			
Hypertension/high blood pressure	18 (43.9)	5 (27.8)	13 (56.5)
Arthritis	16 (39.0)	7 (38.9)	9 (39.1)
Asthma	10 (24.4)	3 (16.7)	7 (30.4)
Diabetes (type 1, type 2, gestational)	9 (22.0)	3 (16.7)	6 (26.1)
Mood disorders (bipolar disorder, cyclothymia, etc.)	7 (17.1)	2 (11.1)	5 (21.7)
Cardiovascular disease (e.g., heart failure, coronary artery disease)	5 (12.2)	0 (0.0)	5 (21.7)
Chronic obstructive pulmonary disease	5 (12.2)	2 (11.1)	3 (13.0)
Neurological conditions (e.g., Parkinson’s disease)	5 (12.2)	1 (5.5)	4 (17.4)
History of stroke	4 (9.8)	1 (5.5)	3 (13.0)
Cancer	2 (4.9)	2 (11.1)	0 (0.0)
Other^d^	19 (46.3)	6 (33.3)	13 (56.5)
None of the above	7 (17.1)	4 (22.2)	3 (13.0)

^a^Missing data included in calculation of percentages

^b^Patients could select more than one choice

^c^Participants could select more than one choice

^d^Other refers to a comorbidity that was described only once and includes, but is not limited to, HIV/AIDS, multiple sclerosis, hypothyroidism, obesity, Hashimoto’s disease, etc

*AIDS *acquired immunodeficiency syndrome; *COVID-19 * coronavirus disease 2019; *HIV* human immunodeficiency virus;* SD* standard deviation

### Concept to item mapping

To analyze the conceptual coverage of the SE-LC19, the items identified were mapped to the subdomains and domains of the conceptual model, resulting in coverage of 34 items (Table 2). The conceptual coverage was generally good and most symptoms were explicitly mapped to one SE-LC19 item.

**Table 2:** Concept-to-item mapping of the SE-LC19

Domain	Sub-domain	SE-LC19	Item(s)
**Upper respiratory tract (5)**	Phlegm	✓	*Phlegm*
	Runny nose and sneezing	✓✓	*Runny nose Sneezing*
	Sore throat	✓	*Sore throat*
	Dry mouth		
	Shortness of breath	≡	*Shortness of breath or difficulty breathing*
**Lower respiratory tract (3)**	Cough	✓	*Cough*
	Chest pain	✓	*Chest pain*
	Difficulty breathing	≡	*Shortness of breath or difficulty breathing*
**Systemic (13)**	Fatigue	✓	*Fatigue*
	Weakness		*Body aches such as muscle pain or joint pain*
	Joint pain	≡	
	Muscle aches and pains	≡	*Body aches such as muscle pain or joint pain*
	Body aches and pains	≡	*Body aches such as muscle pain or joint pain*
	Stiffness		
	Fever	✓	*Fever*
	Chills	✓	*Chills*
	Hot flushes	✓	*Hot flushes*
	Sweats	✓	*Sweats*
	Heart palpitations	✓	*Rapid, strong, or irregular heartbeat*
	Decrease in appetite and weight loss	✓	*Loss of appetite*
	Changes to menstrual cycle		
**Smell and taste (2)**	Altered or loss of taste	✓	*Altered or loss of taste*
	Altered or loss of smell	✓	*Altered or loss of smell*
**Neuro-cognitive (12)**	Numbness	≡	*Pins and needles ornumbness*
	Pins and needles	≡	*Pins and needles or numbness*
	Ringing in ears	✓	*Ringing or buzzing in the ears*
	Dizziness	✓	*Dizziness*
	Lightheaded	✓	*Feeling lightheaded*
	Headache	✓	*Headache*
	Confusion	✓	*Confusion*
	Forgetfulness/memory problems	✓	*Memory problems*
	Brain fog	✓	*Brain fog*
	Concentration	✓	*Loss of concentration*
	Difficulties finding the right word	✓	*Inability to find the right words*
	Challenges with fine motor skills		
**Gastrointestinal (3)**	Difficulty swallowing		
	Nausea	✓	*Nausea*
	Digestive problems	✓✓✓	*Diarrhea* *Vomiting* *Stomachache*
**Other (3)**	Skin changes	✓	*Rash*
	Red or dry eyes	✓	*Red or watery eyes*
	Vision changes		

✓ indicates an explicit match to the item. The number of ✓ corresponds to the number of items mapped to the concept. ≡ indicates that the wording/content of the item maps on two or more sub-domains (e.g., the item pins and needles or numbness matches with BOTH numbness and pins and needles sub-domains). *SE-LC19* Symptoms Evolution of Long COVID-19

### Cognitive debriefing

#### Patient perspectives of the SE-LC19

The instructions of the SE-LC19 were found by the patients to be appropriate and easy to understand. In general, patients were able to understand all 40 items clearly. The 24-h recall period was generally considered by patients to be easier to recall and more convenient to reflect on than a longer time period, such as 7 days. However, some patients mentioned that a 7-day recall period could have enabled them to report more symptoms, as not all symptoms consistently appeared every 24 h. This was of particular relevance as the disease progressed and symptoms were noted to appear more irregularly.

Patients often mentioned that they were uncertain if the symptoms they reported were related to long COVID, as many of the symptoms on the SE-LC19 questionnaire were also common symptoms for other viral infections. Most patients considered the response options for SE-LC19 appropriate. Incidental suggestions included creating a 5-point scale, adding a response to indicate that respondents are uncertain if a symptom should be attributed to long COVID, as well as providing definitions for each response option.

### Conceptual relevance

Overall, patients considered the content of the instrument to be comprehensive and relevant. Four out of 40 items (vomiting, rash, earache, and ringing or buzzing in ears) were not considered relevant by more than 25% of patients.

Minor conceptual gaps or missing content in the SE-LC19 were described by some patients. Some concepts reported spontaneously by a small number of patients included difficulty swallowing, dry mouth, weakness, stiffness, changes to menstrual cycle, challenges with fine motor skills, and vision changes.

Overall, most patients did not report challenges with determining the conceptual overlap and distinctions, except for a few select items. For example, a few patients (n = 3) considered that the item pressure or tightness in chest conceptually overlapped with chest pain. The items confusion (n = 26) and brain fog (n = 34) were considered by some patients to overlap conceptually with each other and other cognitive items such as loss of concentration or memory. Loss of concentration was also considered by 13 patients to conceptually overlap with brain fog. However, several other patients reported confusion and brain fog to be conceptually distinct, though they were unable to provide distinct definitions of each concept. The item pins and needles or numbness was considered by 11 patients to be conflating two separate concepts.

### Meaningful change in response options

During the interview process, patients were asked the following question: “You answered x for the symptom y. How would your answer need to change for it to constitute a meaningful change in symptoms? What change would make a difference in your life?” Patients vocalized meaningful changes between response options (mild, moderate, and severe symptom severity) in one of the following three ways: (1) Change in the intensity or frequency of that symptom, which was reflective of symptom worsening or improving; (2) Change in the daily life or the ability of the participant to engage in activities due to worsening or improvement in the experience of the symptom; and (3) Worsening or improvement, which would be represented by an event such as hospitalization or ability to return to work.

With regards to meaningful improvement for impact on daily life, one patient stated:

“Sometimes my fatigue feels like my body weighs 1000 pounds and I physically… it gets so intense that I can’t lift my head off the pillow. So that could be a change for me would be for me to be able to get up in the morning, maybe even have a glass of water and be able to actually function without just feeling it takes everything I have just to get out of bed…. If I was able to do that I would probably say moderate.” (Female, 50–59 years)

With regards to meaningful deterioration for change in symptom intensity or frequency, one patient stated:

“[Interviewer: Since you chose moderate, what would have to be different about your headaches for you to answer severe instead of moderate?] For me it’d have to interfere… if it caused me to shut down. If I had a headache so severe to where I had to go lay down and I couldn’t do anything.” (Male, 50–59 years)

### Description of item responses of the SE-LC19 questionnaire

**Fig. 1:**
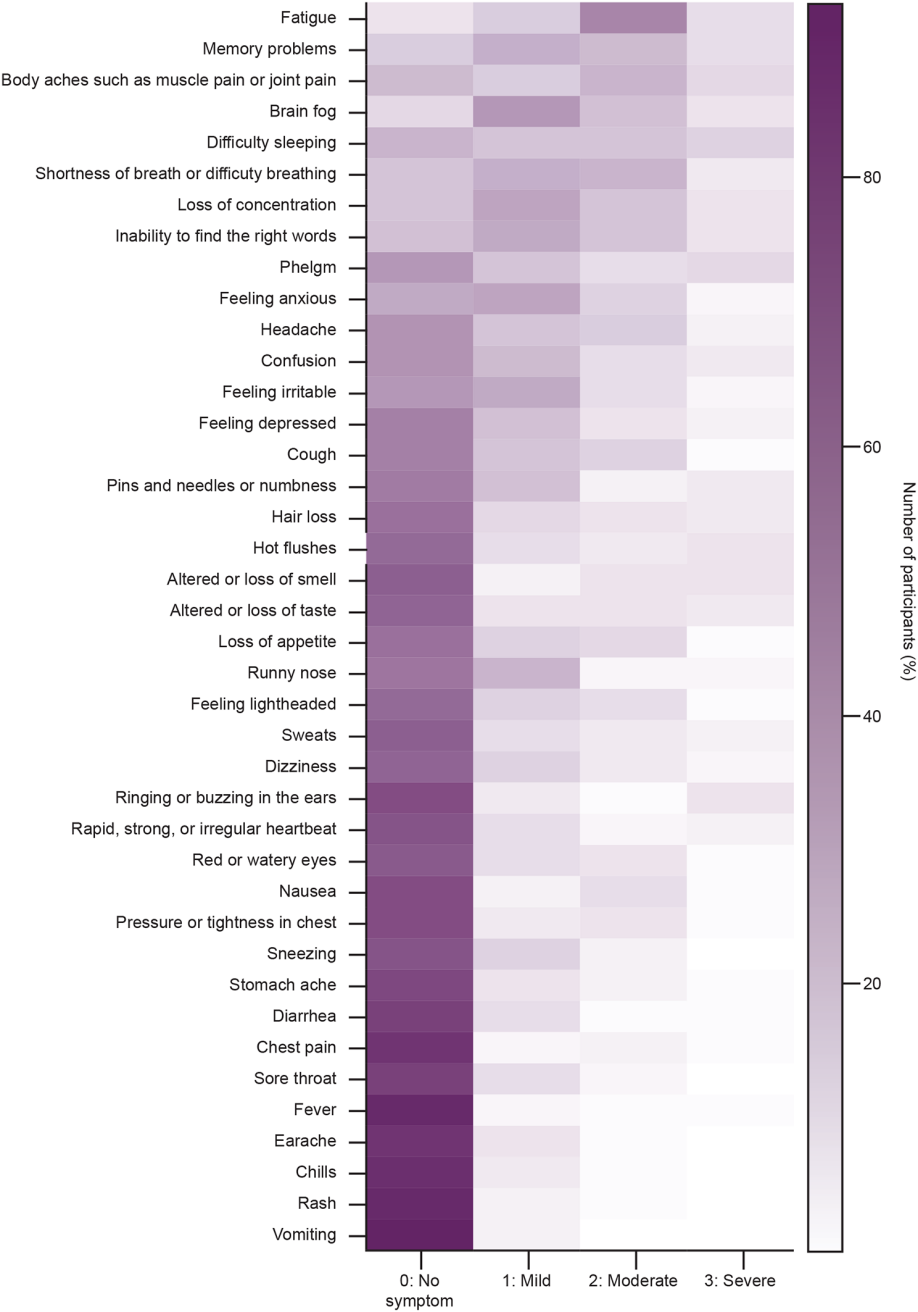
Description of patients’ responses to the items of the SE-LC19 questionnaire. The items are ordered in terms of category frequencies, with darker colors representing higher endorsement percentages. *SE-LC19* Symptoms Evolution of Long COVID-19

The most reported symptoms with moderate severity were fatigue, neuro-cognitive symptoms, body aches, shortness of breath, and difficulty sleeping (Fig. 1). Emotional impacts, such as feeling depressed, were reported in the mild, moderate, and severe categories.

#### Clinician perspectives of the SE-LC19

Similar to the patient interviews, clinicians (n = 5) with different medical specialties (Table S2) found the questionnaire for SE-LC19 very comprehensive. They considered some symptoms, such as shortness of breath, fatigue, and brain fog, to be more relevant than others, such as runny nose, red or watery eyes and hot flashes. The only additional symptom that clinicians suggested was changes to menstrual cycle.

### Exploratory RMT analysis

An initial pooled exploratory RMT analysis of the SE-LC19 items (n = 37) demonstrated that the symptoms of long COVID did not fit together on a single continuum, indicating a requirement for subscales. Items were split into four subscales: neuro-cognitive, systemic, impact, and respiratory. A fifth subscale, most relevant items (symptoms), was tested based on a combination of qualitative results on patients’ most bothersome and frequent symptoms, as well as concepts identified for meaningful improvement. Most of the sample population was covered by the item range (Fig. S1A) for the neuro-cognitive subscale, whereas slight mistargeting was observed for the systemic (Fig. S1B) and respiratory subscales (Fig. S1C), indicating that patients were experiencing less severe symptoms than measured by these subscales. Targeting was adequate for most of the patients for the impact subscale (Fig. S1D), while it was good for the most relevant symptoms subscale (Fig. S1E).

**Fig. 2:**
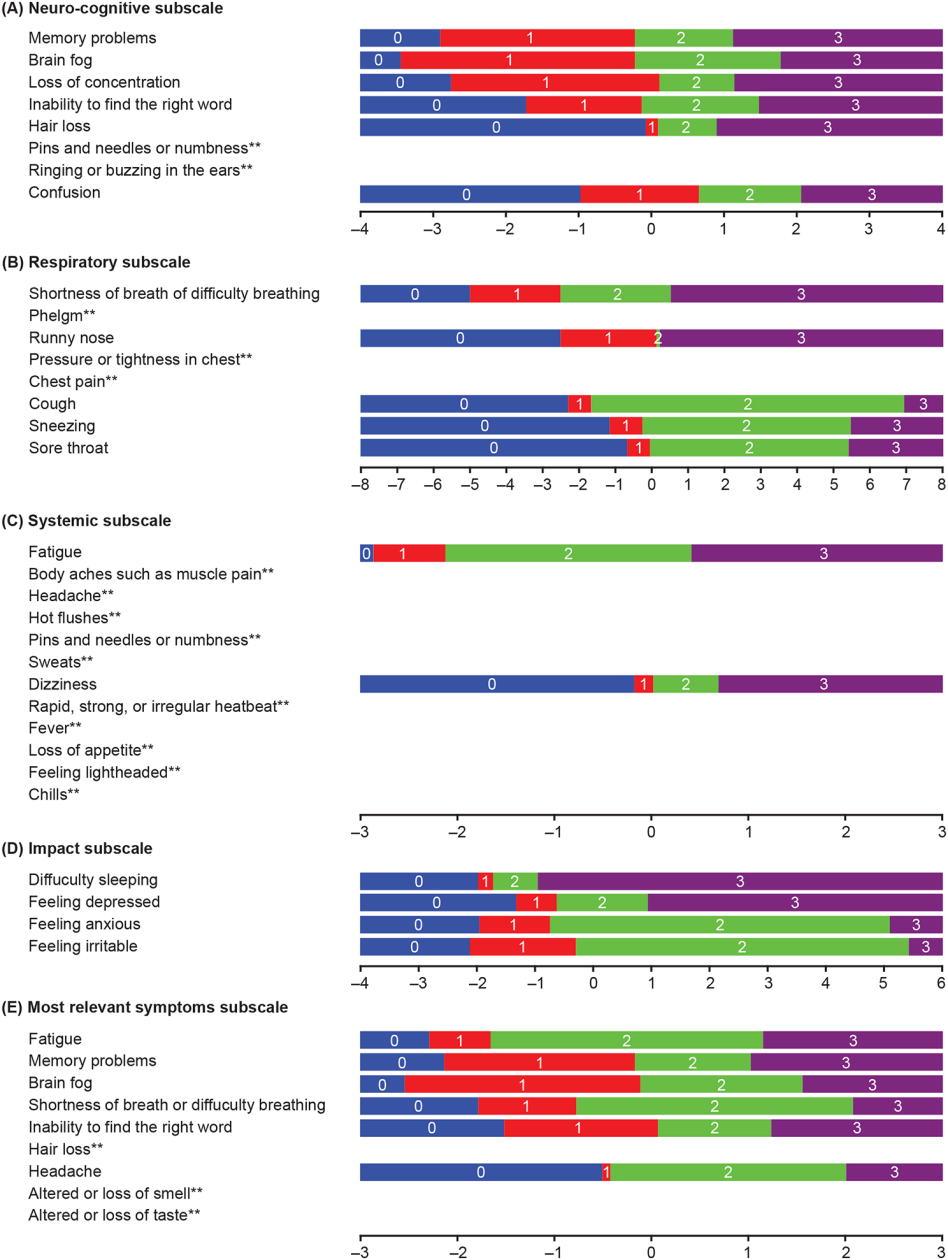
(**A**) Neuro-cognitive subscale, (**B**) respiratory subscale, (**C**) systemic subscale, (**D**) impact subscale, and (**E**) most relevant symptoms subscale thresholds. **Disordered threshold

Item thresholds, item fit, and item dependency were assessed in each subscale to examine the measurement continuum. When item thresholds were assessed, two out of eight items of the neuro-cognitive subscale (Fig. 2A), three out of eight items of the respiratory subscale (Fig. 2B), and 10 out of 12 items of the systemic (Fig. 2C) subscale displayed disordered item response thresholds, indicating that response categories for those particular items were not interpreted in a consistently ordered manner. No items of the impact subscale displayed disordered item response thresholds (Fig. 2D). Three items of the most relevant symptoms subscale also displayed disordered item response thresholds (Fig. 2E). Item fit was very good for the neuro-cognitive, respiratory, impact, and most relevant symptoms subscales (Table S3), as indicated by all fit residuals being within recommended bounds. For the systemic subscale, item misfit was not interpreted as it was affected by the functioning of response categories. When item dependency was examined, no items of the neuro-cognitive, systemic, and impact subscales were found to show potential local dependence. With regards to the respiratory subscale, runny nose and sneezing, and chest pain and pressure or tightness in chest had significant residual correlations. Potential item redundancy was detected between altered or loss of smell and altered or loss of taste in the most relevant symptom subscale. When the samples measurement was assessed, the neuro-cognitive (person separation index [PSI]: 0.85), systemic (PSI: 0.77), respiratory (PSI: 0.85), and most relevant symptoms (estimated PSI: 0.86) subscales had good reliability. The impact subscale had reasonable reliability (estimated PSI: 0.60).

## Discussion

The aim of the present mixed-methods study was to confirm the content validity of the SE-LC19. Through a series of qualitative interviews with patients and clinicians, and exploratory RMT analysis, we found that the SE-LC19 was an easy-to-use instrument that comprehensively measures symptoms evolution in patients with long COVID. The novel instrument may be suitable in monitoring symptoms through the various stages of long COVID, including onset, tracking, and recovery. The SE-LC19 instrument was developed with the extensive involvement of patients with lived experience of long COVID, which is regarded as critical in the development of tools to measure patient-reported outcomes, as well as clinicians experienced in the management of the disease [23].

The content of the 40 items of the SE-LC19 was comprehensive, and while minor conceptual gaps revealed nuances in the experience of some symptoms, the instrument was found to be relevant to the target population of patients with long COVID. The cognitive debriefing of the SE-LC19 confirmed that all of the items were appropriate and understood by most participants. Further research is encouraged to better understand the prevalence of symptoms that were rarely reported by patients in our analysis to understand if they are truly a reflection of long COVID or perhaps other health complexities, especially given the high rate of comorbidities in this study. Exploratory RMT analysis was carried out on a small sample (n = 37) to understand the preliminary psychometrics of the SE-LC19. Overall targeting and item fit were adequate or good for all subscales, and most subscales (neurocognitive, respiratory, impact, and most relevant symptoms) were relevant items being interpreted well by patients. Some items (such as ringing in the ears, and loss of smell and taste) were either present or absent, and systemic symptoms were found to may not be relevant for this population. Overall, exploratory RMT analysis provided evidence that the SE-LC19 questionnaire performed as intended in a patient population with long COVID, however, further research could further investigate the psychometric properties of the SE-LC19 tool using both modern and classical methods.

As mentioned in the introduction, at the time of this study, there were no patient-reported outcome instruments available specific to long COVID. However, the field is rapidly evolving and a number of other assessment tools for long COVID have now been developed [6, 24–27]. The C19-YRS tool was one of the first instruments to be developed specifically for long COVID, capturing symptoms, severity, and functional disability [26]. The first version was based on clinical experience of treating patients with COVID-19 and previous experience of treating related outbreaks [26, 28]. The psychometric properties of the scale were subsequently measured using Rasch analysis, and this, along with emerging information on long COVID, and feedback from patients and professionals, was used to produce a modified version, the C19-YRSm [6]. The scale includes 17 items, distributed between four sub-scales: severity, functional limitations, other symptoms, and overall health. Whilst it has been used on several occasions, it does not contain neurological symptoms, which have been spontaneously mentioned by qualitative research from concept elicitation interviews with patients [12] and highlighted as important to patients with long COVID, as they are specifically burdensome symptoms.

The SBQ-LC scale measures symptom burden in adults with long COVID and was developed at the University of Birmingham using a variety of sources such as published reviews and social media channels for the initial conceptual framework [24]. The scale has been through cognitive debriefing and Rasch analysis, but has not yet been adopted widely in clinical trials and routine care. Little information on the usability of the scale is available but, with 123 items to complete, it may be considered by some as lengthy and burdensome to complete. Furthermore, the authors acknowledge that the validation cohort was not representative and that statements of social media participants, including actual confirmation of COVID-19, could not be confirmed.

The long COVID Symptom Tool and Impact Tool, developed in France, assesses 53 symptoms grouped into ten domains and six aspects of daily life [27]. The severity of symptoms is not captured, only the presence or otherwise, and no information is given as to how long it takes to complete the questionnaire. The authors acknowledge that the instrument has not been tested for longitudinal impact, and that the content validity and clarity were assessed by only two patients with long COVID.

Despite the availability of these other tools since we developed SE-LC19, we believe our tool is the most patient-relevant and comprehensive to date, given that it builds on the foundations of the original COVID-19 work, takes into consideration clinical guidelines, and is content-valid with support for the measure psychometrically. Further research is needed to explore the use of the SE-LC19 in long term studies, and further determine modern and classical psychometrics.

A possible limitation of our research was that participants recruited from the external recruitment agencies initially mirrored the inclusion and exclusion criteria from the clinical trials. This approach may have resulted in a missed profile of patients with long COVID that may be experiencing symptoms differently and/or do not have risk factors for severe COVID-19. Additional interviews may be useful in patients that do not fit the trial inclusion criteria to determine if they experience other symptoms or if the symptom manifestation may somehow be different. At the time of our study, data around long COVID were still limited, and it was not clear how symptoms changed from day to day and how symptoms manifested for patients. While our study showed that most patients found the 24-h recall period acceptable, a couple of patients suggested a longer recall period to capture the waxing and waning of symptoms. Further research could explore the adequacy of a 7-day recall period. Most of the patients in the present study were female (85%) and White (73%). While this is consistent with large epidemiological studies reporting the prevalence of long COVID symptoms [29, 30], a more diverse study population may have identified differences in symptoms between sex and races. In addition, most patients (71%) at the time of the interview reported their general health as good or fair. While six patients (15%) did report their general health as poor and most patients reported some comorbidity, future research could further explore those who specifically rated their overall health as very severe. Finally, it should also be acknowledged that the study’s inclusion/exclusion criteria did not specifically identify patients with myalgic encephalomyelitis/chronic fatigue syndrome, the symptoms and biological abnormalities of which may overlap with long COVID [31].

As the burden of long COVID on healthcare systems grows, instruments, like SE-LC19, will become essential to monitor the progress of patients and to direct the most appropriate resources to meet demand. This mixed-methods research provides supportive evidence of the content validity of the SE-LC19 to assess the symptoms of patients with long COVID. Future research is needed to further develop a scoring algorithm, determine the psychometric properties, and determine meaningful within-patient changes. Robust, patient-relevant, meaningful endpoints are needed to support decision-making with various stakeholders. Further, longitudinal, and real-world studies are also recommended to document the pattern of long COVID experienced in a larger, more diverse study population.

## Conclusion

This mixed-methods study provides evidence for the content validity of the SE-LC19 instrument to evaluate the symptoms of patients with a confirmed diagnosis of long COVID. Overall, patients and clinicians found the instructions, recall period, and questions relevant and applicable to the disease, and support the use of SE-LC19 in clinical practice, and future cross-sectional and longitudinal clinical studies specifically investigating long COVID. Further research is warranted to explore the psychometric properties of the instrument to further solidify that it is fit for purpose in measuring symptoms and severity of long COVID in daily clinical practice, as well as research and clinical trial settings.

### Electronic supplementary material

Below is the link to the electronic supplementary material.

Supplementary Material 1

## Data Availability

Not applicable.

## Abbreviations

CDC: Centers for Disease Control and Prevention
COVID-19: Coronavirus disease 2019
FDA: Food and Drug Administration
IRB: Institutional Review Board
NICE: National Institute for Health and Care Excellence
PCC: Post-COVID conditions
PCS: Post-COVID-19 syndrome
PSI: Person separation index
RMT: Rasch Measurement Theory
SARS-CoV-2: Severe acute respiratory syndrome coronavirus 2
SD: Standard deviation
SE-C19: Symptoms Evolution of COVID-19
SE-LC19: Symptoms Evolution of Long COVID-19
WCG-IRB: Western Institutional Review Board and Copernicus Group Institutional Review Board
WHO: World Health Organization
WIRB: Western Institutional Review Board
