# Supplementary material for: The symptoms evolution of long COVID‑19 (SE-LC19): a new patient-reported content valid instrument
Source: J Patient Rep Outcomes. 2024 Aug 9;8:87. doi: 10.1186/s41687-024-00737-5 (PMC11310370; doi:10.1186/s41687-024-00737-5)
Supplement: Supplementary file 1 — Supplementary Material 1 [file 41687_2024_737_MOESM1_ESM.docx]

# Supplementary Material

## Supplementary methods

### Participant eligibility criteria

Participants were eligible for inclusion in the study if they met the following criteria:

- Willing and able to provide electronic informed consent.
- ≥18 years old.
- Experiencing known symptom(s) of long COVID that cannot be explained by an alternative diagnosis.
- Willing and able to participate in a 60-minute audio recorded one-on-one interview.
- Able to speak, read, write, and understand English.
- Has access to the internet (e.g., computer or telephone app) to complete an electronic consent form and to participate in the interview.
- Was not vaccinated at the time of COVID-19 infection or 90 days thereafter.
- The initial COVID-19 infection was at least 180 days prior.
- Must have received their final dose (including booster shots) of the COVID-19 vaccination at least 4 weeks prior to scheduling the interview.
- Must have ≥1 risk factor for severe COVID-19. Risk factors are defined as follows:
  - Age ≥50 years.
  - Obesity, defined as body mass index (BMI) ≥30 kg/m^2^ OR
  - Cardiovascular disease, including hypertension
  - Chronic lung disease, including asthma
  - Type 1 or type 2 diabetes mellitus
  - Chronic kidney disease, including those on dialysis
  - Chronic liver disease
  - Immunosuppressed because of treatments received or pre-existing conditions (e.g., cancer treatment, bone marrow or organ transplantation, immune deficiencies, HIV (if poorly controlled or evidence of AIDS), sickle cell anemia, thalassemia, and prolonged use of immune-weakening medications).
- Participants recruited from agencies (not Regeneron Pharmaceutical, Inc. clinical trials) were required to complete a clinician-signed confirmation of diagnosis form that stated that the symptoms could not be explained by an alternative diagnosis in the clinician’s opinion.

Participants meeting the following criteria were excluded from the study:

- Concomitant illness(es) that in the recruiter or investigator’s judgment would have confounded the researchers’ understanding and description of their long COVID disease experience.

### Exploratory RMT analyses

Exploratory RMT analysis was used to enable the development of an equal interval measurement scale that works the same for all patients, regardless of their ability level. Targeting within RMT was evaluated through the examination of the relative person and item threshold histograms. Person and item location histograms were plotted against the same measurement continuum on the log-odds (logit) scale. The relative distributions displaying the person and item locations informed the adequacy of the sample for evaluating the scale and the adequacy of the scale for measuring the sample. A scale with adequate targeting was expected to have an item distribution that spans the full range of person locations. A sample with adequate targeting was expected to have a distribution that closely matches the item distribution. A comparison of the person and item mean locations was also used to assess targeting. The item location mean was always set at zero logits. A person location mean proximal to the item mean indicated adequate targeting. A mistargeted scale or one with inconsistent coverage of the sample might fail to pick up changes in person locations.

For analyses of item thresholds during the evaluation of the measurement continuum, the use of response categories for each item scored with successive integer scores (e.g., 0, 1, 2, 3, 4) hypothesized a continuum of increasing impact, from less to more, in terms of the measured subdomain. This hypothesis was tested by examining the ordering of thresholds (or points of crossover between two adjacent response categories) ascertained by the exploratory RMT analysis. A threshold is the point on the measurement continuum at which the probability of responding to adjacent categories (e.g., ‘mild’ and ‘moderate’) is equal. Disordered thresholds imply scoring functions that are not working as intended. In addition, item and threshold maps may help provide a sense of symptom progression. Tests of local dependency LD were done to determine whether the response to any item directly impacted any other item in the subscale. RMT analysis determines this effect by examining the residual correlations. Item pairs with potential dependence were flagged.

The RMT scale items were expected to work well together clinically and statistically to determine whether items defined a cohesive continuum. Statistical indicators of item cohesiveness examined the goodness-of-fit of the observed rating scale data to the requirements of the RMT model. Fit residual estimates were recommended to lie between +2.5 and –2.5. Chi-square values summarized the difference between the observed mean scores for each item within groups of people (class intervals) with similar levels of the construct under measurement with the scores expected by the RMT model. Significant chi-square values indicated differences between observed scores and expected values that exceeded chance expectations.

### **Table S1** SE-LC19 questionnaire items

| **SE-LC19 questionnaire items** | | | |
| --- | --- | --- | --- |
| Fever | Ringing or buzzing in the ears | Feeling lightheaded | Rash |
| Cough | Sore throat | Diarrhea | Red or watery eyes |
| Phlegm | Altered or loss of taste | Nausea | Difficulty sleeping |
| Shortness of breath or difficulty breathing | Altered or loss of smell | Vomiting | Pins and needles or numbness |
| Chills | Loss of appetite | Dizziness | Feeling irritable |
| Fatigue | Confusion | Pressure or tightness in chest | Feeling depressed |
| Headache | Brain fog | Chest pain | Feeling anxious |
| Runny nose | Loss of concentration | Rapid, strong or irregular heartbeat | Hair loss |
| Sneezing | Memory problems | Body aches such as muscle pain or joint pain | Sweats |
| Earache | Inability to find the right words | Stomach ache | Hot flushes |

### Table S2 Overview of clinician sample characteristics

| **Current position(s)** | **Specialty/training** | **Number of years practicing** | **Number of long COVID-19 patients treated per week** |
| --- | --- | --- | --- |
| Attending physician at academic medical center | Pulmonary and critical care | 30 years | 40 |
| Solo private practice | General medicine | 20 years | 20 |
| Attending physician in emergency department | Emergency medicine | 15 years | Missing |
| Registered nurse at a hospital | Registered nurse | 30 years | 10–20 |
| Registered nurse at a hospital | Registered nurse | 26 years | Missing |

### **Table S3** Evaluation of item fit for SE-LC19 subscales

| **Item** | **Statement** | **Location** | **Standard error** | **Fit residual** | **Chi^2^** | **Probability** |
| --- | --- | --- | --- | --- | --- | --- |
| **Neurocognitive subscale** | |  |  |  |  |  |
| SE11 | Ringing or buzzing in the ears | 0.573 | 0.234 | –0.221 | 1.653 | 0.4376 |
| SE16 | Confusion | 0.604 | 0.269 | –0.983 | 3.337 | 0.1886 |
| SE17 | Brain fog | –0.612 | 0.286 | –1.066 | 3.381 | 0.1844 |
| SE18 | Loss of concentration | –0.479 | 0.264 | –1.33 | 1.807 | 0.4051 |
| SE19 | Memory problems | –0.65 | 0.263 | –0.45 | 1.197 | 0.5497 |
| SE20 | Inability to find the right words | –0.106 | 0.255 | 1.378 | 2.213 | 0.3306 |
| SE34 | Pins and needles or numbness | 0.348 | 0.239 | 1.908 | 3.735 | 0.1545 |
| SE38 | Hair loss | 0.322 | 0.235 | 2.062 | 1.361 | 0.5063 |
| **Respiratory subscale** | |  |  |  |  |  |
| SE2 | Cough | 1.018 | 0.287 | –0.136 | 2.852 | 0.2403 |
| SE3 | Phlegm | –2.073 | 0.238 | 0.235 | 4.076 | 0.1303 |
| SE4 | Shortness of breath or difficulty breathing | –2.305 | 0.312 | 0.093 | 0.332 | 0.8471 |
| SE8 | Runny nose | –0.695 | 0.315 | –0.490 | 1.930 | 0.3809 |
| SE9 | Sneezing | 1.399 | 0.354 | –0.238 | 0.589 | 0.7450 |
| SE12 | Sore throat | 1.595 | 0.387 | 0.214 | 1.091 | 0.5795 |
| SE26 | Pressure or tightness in chest | 0.350 | 0.292 | –0.275 | 0.826 | 0.6617 |
| SE27 | Chest pain | 0.711 | 0.358 | –0.589 | 0.623 | 0.7322 |
| **Impact subscale** | |  |  |  |  |  |
| SE33 | Difficulty sleeping | –1.525 | 0.2 | –0.267 | 0.853 | 0.6529 |
| SE35 | Feeling irritable | 1.022 | 0.288 | 0.78 | 0.072 | 0.9645 |
| SE36 | Feeling depressed | –0.308 | 0.258 | –0.022 | 1.643 | 0.4398 |
| SE37 | Feeling anxious | 0.811 | 0.271 | 1.607 | 0.326 | 0.8494 |
| **Most relevant symptoms subscale** | | |  |  |  |  |
| SE6 | Fatigue | –0.92 | 0.246 | 0.546 | 0.567 | 0.7533 |
| SE7 | Headache | 0.377 | 0.236 | 0.128 | 0.509 | 0.7754 |
| SE13 | Altered or loss of taste | 0.633 | 0.229 | –0.763 | 3.445 | 0.1787 |
| SE14 | Altered or loss of smell | 0.509 | 0.214 | –0.355 | 0.6 | 0.7407 |
| SE17 | Brain fog | –0.343 | 0.256 | –0.833 | 2.442 | 0.2950 |
| SE19 | Memory problems | –0.414 | 0.238 | 0.279 | 4.527 | 0.1040 |
| SE20 | Inability to find the right words | –0.057 | 0.237 | 0.362 | 0.598 | 0.7414 |
| SE38 | Hair loss | 0.366 | 0.221 | 1.837 | 1.259 | 0.5328 |
| **Systemic subscale** | |  |  |  |  |  |
| SE1 | Fever | 0.228 | 0.263 | 1.512 | 21.455 | <0.0001 |
| SE5 | Chills | 2.01 | 0.448 | –0.459 | 0.864 | 0.6492 |
| SE6 | Fatigue | –1.511 | 0.24 | 0.382 | 0.804 | 0.6689 |
| SE7 | Headache | -0.398 | 0.209 | –0.163 | 0.853 | 0.6526 |
| SE15 | Loss of appetite | 0.344 | 0.237 | –0.616 | 0.851 | 0.6536 |
| SE21 | Feeling lightheaded | 0.654 | 0.243 | 0.057 | 0.74 | 0.6907 |
| SE25 | Dizziness | 0.19 | 0.243 | –1.083 | 4.683 | 0.0962 |
| SE28 | Rapid, strong, or irregular heartbeat | 0.196 | 0.25 | –0.963 | 1.461 | 0.4816 |
| SE29 | Body aches such as muscle pain or joint pain | –1.045 | 0.192 | 0.28 | 0.104 | 0.9493 |
| SE34 | Pins and needles or numbness | –0.287 | 0.218 | –0.392 | 2.152 | 0.3410 |
| SE39 | Sweats | –0.005 | 0.232 | 0.712 | 1.695 | 0.4286 |
| SE40 | Hot flushes | –0.376 | 0.197 | 0.394 | 1.449 | 0.4846 |

Recommended range for fit residuals is –2.5 to +2.5.

### Fig. S1: Scale-to-sample targeting for (A) neuro-cognitive subscale, (B) systemic subscale, (C) respiratory subscale, (D) impact subscale, and (E) most relevant symptoms subscale

**A**


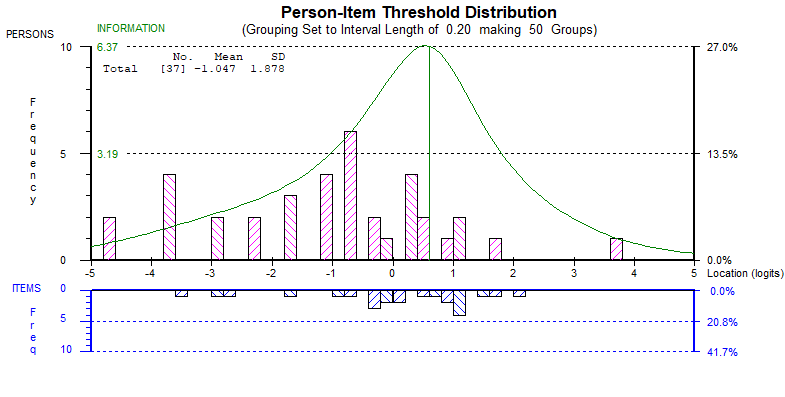


**B**


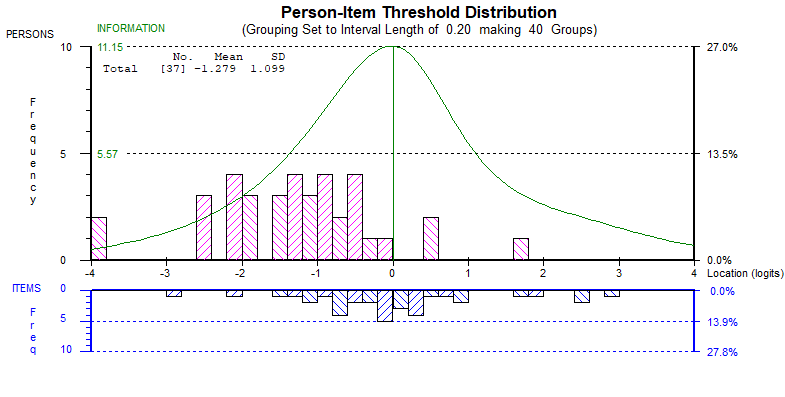


**C**


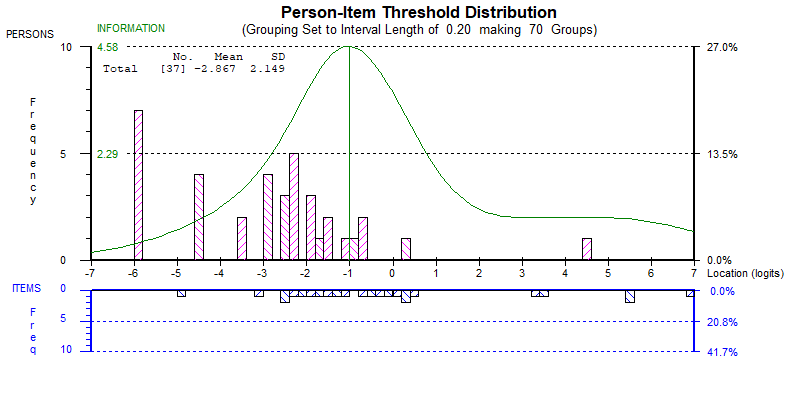


**D**


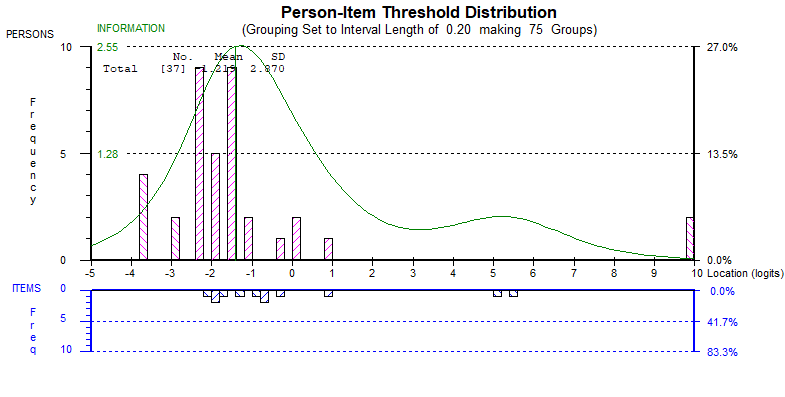


**E**


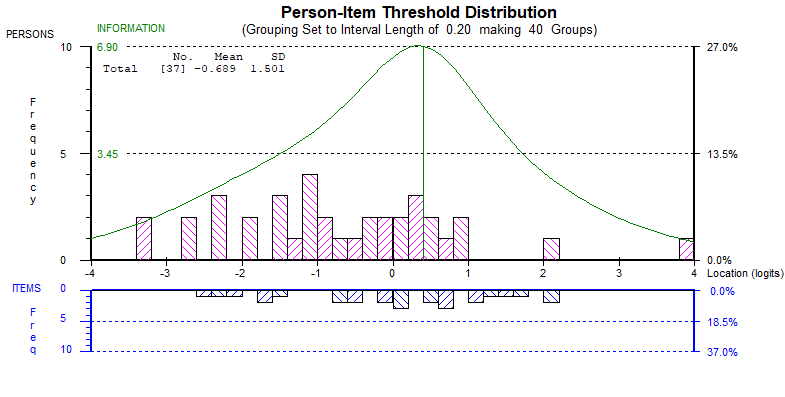


*SD* standard deviation.
